# Miscellaneous

**Published:** 1891-12

**Authors:** 


### MISCELLANEOUS.

### MUSH AND MILK.

A famous doctor says : Eat a good bowl of mush and milk for your breakfast and you will not need any medicine.

Indian corn contains a large amount of nitrogen, has qualities anti-constipating, and is easily assimilated. It is cheap and has great nutritive properties. A course of Indian meal in the shape of Johnny-cake, hoe-cake, corn or pone bread and mush, relieved by copious draughts of pure cow's milk, to which, if inclined to dyspepsia, a little lime water may be added, will make a life now a burden well worth the living, and you need no other treatment to correct your nervousness, brighten your vision, and give you sweet and peaceful sleep.

### TEA POISONING.

Russian tea merchants, who, when present on the Chinese frontier for buying tea, are, it is said, obliged to taste from one hundred and fifty to two hundred specimens of strong tea infusions daily, do not swallow the infusion, but, nevertheless, a slow intoxication appears in them. The symptoms are loss of appetite, constipation which alternates with diarrhœa, failure of general nutrition, periodical epigastric pains, enlargement of the liver, with subsequent atrophic cirrhosis, and dryness and sallowness of the skin, hypochondriacal frame of mind, marked failure of memory, of sight, of visual vacuity, and sometimes of taste and smell.

### HOW TO GIVE NITRE.

Sweet spirit of nitre is one of the most popular domestic medicines. The dose for an adult is from one-half to one teaspoonful well diluted with water. When using it in fevers, it is best to give small doses and repeat them often, rather than give large doses at long intervals. One-half a teaspoonful in a tumblerful of cold water, drunk a little at a time through the night, will be more effective in subduing fever and bringing on perspiration than a whole teaspoonful taken at once.

To stimulate the kidneys to greater activity, it is best to use larger doses, and from one-half to one teaspoonful, properly diluted, may be given every three or four hours, until two or three doses have been taken. The continued use of this agent, notwithstanding its simple character, is likely to do harm.

### THE SICK ROOM.

In waiting upon invalids many people make the mistake of serving a quantity of food at a time, thereby taking away the little appetite the sick ones possess. Variety in food should be provided, and only a little delicate portion served at once, so to tempt the appetite. It is always advisable to keep beef-tea, jelly, custards, etc., in readiness, for in many cases if a patient has to wait while these dishes are being prepared, he or she loses the desire to eat, and will often turn from them when brought. In serving food to invalids every thing should look as tempting as possible—a pretty, clean tray cloth, clean cups and saucers, sparkling glass, every thing to invite the appetite. Another great mistake frequently made is the habit of keeping plates of food in the sick room—this should never be done. When something is brought for which the patient has no appetite and refuses to eat, it should be taken away at once and brought again at the right time ; for food left standing by nauseates the invalid, and oftentimes causes him or her to turn from it altogether. The introduction of flowers in the sick room brightens it up as nothing else does, and to the invalid who is confined to the room their sweet scent is grateful and refreshing.

Baron Sir Ferd. Von Mueller, M.D., F.R.S., has introduced in Victoria the use of green eucalyptus branches in sick rooms, by recommending the placing of them under the bedsteads, and renewing them when necessary. He considers this plan applicable to all infectious and contagious diseases, and it is said to have been successful with phthisical patients, not only antiseptically, but also as a sedative, and to some extent hypnotic. Dr. J. B. Curgenven states as his experience of this plan in scarlet fever, in twelve months' trial, that the bedding is thoroughly disinfected, and the volatile vapor penetrates every article, even the mattress, and the room requires no other disinfection, as every germ that escapes from the patient is killed by the vapor.

### WALKING.

Few things, if any, are so effectual in building up and sustaining the physical organization as walking, if resolutely and judiciously followed. It is perfect exercise. It taxes the entire system. When you walk properly every member and muscle, every nerve and fibre, has something to do. What is the effect ? The flesh is solidified ; the lungs grow strong and sound ; the chest enlarges ; the limbs are rounded out ; the tendons swell and toughen ; the figures rise in height and dignity, and is clothed with grace and suppleness. The whole man is developed, not the body merely. The mind is broadened by the contemplation of creation's works, the soul is enlarged, the imagination brightened, the spirits cheered, the temper sweetened.

